# Determining the Need for Metastatic Staging in Patients with Bilateral Breast Cancers

**DOI:** 10.3390/curroncol31040145

**Published:** 2024-04-02

**Authors:** Veronica Siton Alcantara, Sut Mo Zachary Chan, Fuh Yong Wong, John Carson Allen, Geok Hoon Lim

**Affiliations:** 1 Breast Department, KK Women’s and Children’s Hospital, Singapore 229899, Singapore; 2 Division of Radiation Oncology, National Cancer Centre, Singapore 168583, Singapore; 3 Duke-NUS Medical School, Singapore 169857, Singapore; john.allen@duke-nus.edu.sg

**Keywords:** metastatic staging, breast cancer, bilateral cancers, metachronous cancers, systemic metastasis

## Abstract

**Introduction:** Bilateral breast cancers (BBC) diagnosed at an interval apart are uncommon. While metastatic staging guidelines are established in patients with unilateral breast cancer, its role in BBC diagnosed at an interval apart is unclear. We aim to identify the subgroup who would benefit from metastatic staging at contralateral cancer diagnosis. **Methods:** Eligible patients were divided into three categories: (A) ipsilateral invasive cancer and contralateral ductal carcinoma in situ (DCIS), (B) bilateral invasive cancers and (C) ipsilateral DCIS and contralateral invasive cancer and reviewed retrospectively. We excluded patients with bilateral DCIS, synchronous BBC diagnosed within 6 months from first cancer, patients who were stage IV at first cancer diagnosis and patients with recurrence prior to contralateral cancer. **Results:** Of 4516 newly diagnosed breast cancer patients, 79 patients were included. Systemic metastasis occurred in 15.6% of patients in Group B. Having nodal positivity of either cancer which were diagnosed ≤30 months apart and nodal positivity of only the contralateral cancer when diagnosed >30 months apart was significantly associated with systemic metastasis (*p* = 0.0322). **Conclusions:** Both the nodal status and a 30 months cut-off time interval between the two cancers can be used to identify patients who will benefit from metastatic staging. This finding requires validation in larger studies.

## 1. Introduction

Bilateral breast cancer (BBC) is uncommon and has a reported incidence of 1.4–11.8% [1]. In women diagnosed with a primary breast cancer, there is a two to six-fold risk of developing cancer in the contralateral breast [1]. BBC can be further categorized, based on the time interval between the primary and contralateral breast cancer diagnosis, into synchronous BBC or cancers which occurred at an interval apart, with metachronous bilateral breast cancers (MBBC) usually referred to as the group with bilateral invasive cancers. The time interval used for defining synchronous bilateral cancers however varies from one month [2] to a year [3], depending on the definition used in various studies. Nonetheless, synchronous bilateral breast cancer was reported to occur in 1.6 per 105 person-years at risk while MBBC had a higher incidence rate of 440 per 105 person-years at risk [4].

Patients with MBBC were also reported to have a different prognosis from patients with unilateral breast cancer. MBBC patients with contralateral cancer diagnosed within five years of primary cancer were found to present more commonly with distant metastasis compared to unilateral breast cancer [5]. On the other hand, MBBC patients with contralateral cancer diagnosed more than five years apart, had a similar prognosis as unilateral breast cancer [4]. In another study [6], patients with MBBC were found to have a worse disease-free survival compared to patients with unilateral breast cancer. However, the overall survival was similar for both groups of patients. It was also established that the second contralateral breast cancer could sometimes be a metastasis from the first cancer rather than another new primary cancer, with the former group having a poorer prognosis [7]. In such cases, genomic profiling could help distinguish between the two entities, hence providing appropriate treatment [8].

Since contralateral cancer can occur months to years after the primary cancer, it is crucial to distinguish this group of patients from recurrence. While the guidelines for metastatic staging are established in patients with unilateral breast cancer [9] and in patients with recurrence [10], the role of metastatic staging in patients with BBC diagnosed at an interval apart is not well defined. We aimed to determine the risk factors for systemic metastasis in patients with BBC diagnosed at an interval apart, specifically in MBBC, thereby identifying the subgroup of MBBC patients who would benefit from metastatic staging at the time of contralateral cancer diagnosis.

## 2. Materials and Methods

BBC patients who received treatment at KK Women’s and Children’s Hospital, Republic of Singapore, from 1 September 2005 to 30 June 2022 were included in this retrospective study. We excluded patients with bilateral ductal carcinoma in situ (DCIS), patients with bilateral cancers diagnosed at ≤6 months apart, patients with malignant phyllodes or sarcomas, and patients who were diagnosed with stage IV disease at first cancer presentation. Patients who declined treatment at first cancer diagnosis or with evidence of recurrence prior to contralateral cancer were excluded as well.

The patients were categorized into three groups: (A) ipsilateral invasive cancer and contralateral ductal carcinoma in situ (DCIS), (B) bilateral invasive cancers (MBBC) and (C) ipsilateral DCIS and contralateral invasive cancer respectively and reviewed retrospectively.

At our institution, after each biopsy-proven invasive breast cancer diagnosis, metastatic staging which comprised of CT and bone scans would be performed routinely for patients with nodal involvement. However, metastatic staging may also be performed in patients with no nodal involvement, based on the treating physician‘s clinical assessment. In patients with contraindications to the CT scan, a chest X-ray and hepatobiliary ultrasound would be performed instead. Imaging of the brain was usually reserved for patients with neurological symptoms since metastasis to the brain tends to occur more frequently in patients with certain subtypes such as the triple negative or HER2 positive subtypes [11].

Patients with incomplete or no metastatic staging at the contralateral cancer diagnosis, particularly when the contralateral cancer was DCIS, were reviewed on a case-by-case basis for evidence of systemic metastasis. For the purpose of this study, if there was no evidence of systemic metastasis within two years of treatment following the contralateral cancer diagnosis in this group of patients, then these patients would be considered as having no systemic metastasis. It was assumed that the metastatic staging in such patients, if performed at the time of cancer diagnosis, was negative.

Patients with indeterminate findings from metastatic staging performed at contralateral cancer diagnosis were routinely followed up to determine the significance of these indeterminate findings. In this study, these indeterminate findings were classified as benign if there was no evidence of progression or/and recurrence after two years of follow up [12]. Patients with indeterminate findings on the metastatic staging at diagnosis and had a follow up shorter than two years were excluded from this study.

Patient demographics, pathological characteristics of bilateral cancers, and metastatic staging outcomes at contralateral cancer diagnosis were collected from a prospectively maintained database. The various demographic and pathological characteristics were then compared and analysed between patients with systemic metastasis versus those without, to identify the risk factors associated with the group who would require metastatic staging.

The study was approved by SingHealth Centralised Institutional Review Board (CIRB Ref: 2019/2419) and informed consents were waived by the ethics committee.

To identify the risk factors associated with systemic metastasis in MBBC patients (group B), Fisher’s exact test was used to compare the incidence of systemic versus non-systemic metastasis in MBBC patients against various potential categorical risk factors obtained from the patient demographic and pathological characteristics. *p* ≤ 0.05 was defined as statistically significant. SAS statistical software (v9.4) was used for the analysis.

## 3. Results

In total, 4516 patients were newly diagnosed with breast cancer over the study period. Of these patients, 260 developed bilateral breast cancers. After excluding 158 bilateral breast cancer patients with time interval ≤6 months between cancers, 102 patients developed BBC >6 months apart, resulting in a prevalence rate of 2.3% in our cohort. Of the 102 BBC patients, 23 were excluded for the following reasons. A total of four patients had bilateral DCIS, six had stage IV at first cancer diagnosis, two defaulted treatment for their first cancer, five developed recurrence after first cancer prior to contralateral cancer, five had no metastatic staging at contralateral breast cancer diagnosis and were followed up for less than two years and one had indeterminate metastatic staging results with inadequate follow up of less than two years.

**Figure 1:**
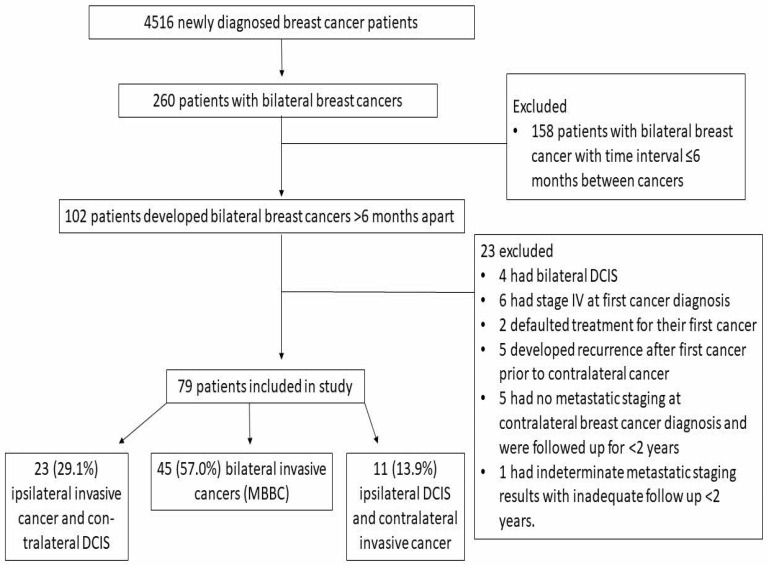
Flowchart of patients in the study.

After excluding these 23 patients, 79 were included in the analysis. A total of 23 (29.1%), 45 (57.0%) and 11 (13.9%) had ipsilateral invasive cancer and contralateral DCIS, bilateral invasive cancers (MBBC) and ipsilateral DCIS and contralateral invasive cancer, respectively (Figure 1). Collectively, the mean age at first and contralateral cancer was 53.0 (range: 30–78) and 58.8 years old (range: 38–82) respectively. The mean time interval between diagnosis of the two cancers was 68.5 months (range: 7–175).

For the 23 patients with ipsilateral invasive cancer and contralateral DCIS, the mean age at first and contralateral cancer was 51.5 (range: 38–71) and 57.1 (range 40–75) years old, respectively [Table 1]. The mean tumour size of the first invasive cancer was 21.6 mm (range: 0.5–62). The mean time interval between the first invasive and contralateral DCIS diagnosis was 68.3 months (range: 11–140).

For the MBBC group, mean age at first and contralateral cancer diagnosis was 53.7 (range: 30–78) and 59.7 years old (range 38–82) respectively [Table 2]. Mean invasive tu-mour size of primary and contralateral cancers was 24.8 mm (range: 1.8–90 mm) and 13.0 mm (range: 1.1–75 mm) respectively. The mean time interval between diagnoses of the two cancers was 71.4 months (range: 7–175 months).

For the ipsilateral DCIS and contralateral invasive cancer group, mean age at first and contralateral cancer diagnosis was 53.8 (range: 41–68) and 58.7 years old (range 43–71), respectively [Table 3]. The mean invasive tumor size of contralateral cancer was 17.6 mm (range: 1.5–50 mm). The mean time interval between diagnoses of the 2 cancers was 57.1 months (range: 11–150 months).

Seven (8.9%) patients had systemic metastasis at contralateral cancer diagnosis, of which all occurred in the MBBC group, resulting in 15.6% of MBBC group with systemic metastasis.

Of the characteristics analyzed for association with systemic metastasis in the MBBC patient group (B), only a combination of nodal status and a time interval of 30 months between cancer diagnosis (combined nodal status) was statistically significant (*p* = 0.0322) [Table 4]. Combined nodal status was defined as ‘positive’ if either the first or the contralateral cancer had nodal metastasis and were diagnosed ≤30 months apart. If diagnosed >30 months apart, combined nodal status was declared ‘positive’ only if the contralateral cancer had nodal metastasis; otherwise, combined nodal status was declared ‘negative’. More succinctly, for cancers diagnosed ≤30 months apart, nodal positivity of either cancer was predictive for systemic metastasis. Conversely, in patients with cancers diagnosed >30 months apart, only the contralateral cancer nodal positivity was associated with systemic metastasis.

## 4. Discussion

BBC that occurred >6 months apart developed in 2.3% of our breast cancer cases, which was consistent with existing literature [4,13]. For BBC patients who met the inclusion criteria for this study, 8.9% had systemic metastasis upon diagnosis of contralateral breast cancer, all of which occurred in the MBBC group. In the MBBC group, nodal positivity of either cancer when they were diagnosed ≤30 months apart and nodal positivity of the contralateral cancer when diagnosed >30 months apart was associated with systemic metastasis.

In our study, synchronous BBC was excluded. Various time intervals ranging from 1 month to one year have been described to distinguish synchronous BBC [14] from MBBC, and this time interval remains controversial. Based on the various time intervals used, conflicting characteristics of MBBC have been described. In a study by Senkus et al. that used a three-month cut-off, it was reported that MBBC had a more aggressive phenotype on immunohistochemical analysis than synchronous bilateral breast cancer [15]. However, another study by Ibrahim et al., found that synchronous BBC defined as presenting within a year from diagnosis of the initial breast cancer, may have a worse prognosis compared to MBBC [16]. In contrast, in a study comparing MBBC with synchronous BBC and unilateral breast cancer by Londero et al., which used a six-month cut-off time, there was no difference in terms of overall survival and disease-free survival between MBBC and synchronous breast cancer [17]. In our study, a six-month interval was used since any contralateral breast lesion would be further evaluated or followed up with surveillance imaging within this duration at our institution. This would hence reduce the risk of any undetected synchronous breast cancer being misclassified as MBBC.

It was also important to distinguish between MBBC and primary tumor recurrence, though the latter tended to be systemic or confined to the ipsilateral side [18]. Nonetheless, in a study by Rubino et al. which distinguished early and late contralateral breast cancer, it was reported that early contralateral breast cancer could still be a possible spread from the primary cancer if it was diagnosed within two years [19].

The indication of metastatic staging in BBC patients diagnosed at an interval apart remained unclear. Bilateral breast cancers were generally more likely to have metastatic disease compared to unilateral breast cancer [20]. It was also reported that the time interval between primary breast cancer and contralateral breast cancer in MBBC can influence the patient’s prognosis, with a longer period between the two cancers being more favourable. MBBC diagnosed after five years from the initial tumor had been reported to have a similar overall survival as unilateral carcinoma [3].

In our study, there was no systemic metastasis noted on the staging for the groups with (1) ipsilateral invasive cancer and contralateral ductal carcinoma in situ (DCIS) and (2) ipsilateral DCIS and contralateral invasive cancer. In the former group, this finding was not surprising since DCIS is preinvasive [21,22]. Nodal involvement [23] and distant metastasis in DCIS are rare. In addition, majority of distant metastasis in invasive cancers usually occurred within the first five years of treatment of the primary invasive cancer in unilateral cancers [24]. In BBC, more than half of patients who developed contralateral breast cancer within three years from the primary cancer developed distant metastasis [25]. Since our average time interval between the first invasive and contralateral DCIS was longer than five years, metastatic staging may not be indicated in this group unless there were clinical indications.

In the latter group with ipsilateral DCIS and contralateral invasive cancer, a prior diagnosis of DCIS should not affect the decision for metastatic staging since DCIS is preinvasive. Unfortunately, this group had a small sample size. Hence, although no metastatic disease was detected on staging, the use of metastatic staging at the contralateral invasive cancer diagnosis should be carried out in accordance with the guidelines for unilateral breast cancer for this group of patients.

For the MBBC group, nodal status combined with time interval between the cancers was predictive of systemic metastasis. This was not surprising since lymph node metastasis was known to be a poor prognostic factor in unilateral breast cancer [26,27] and was associated with distant metastasis [28]. In patients with MBBC, lymph node metastasis at contralateral breast cancer diagnosis was similarly also associated with a poorer prognosis [29]. As a result, metastatic staging was advocated in patients with unilateral breast cancer when they have advanced disease or with symptoms of systemic metastasis. Similarly in synchronous BBC, metastatic staging could be reserved for patients with nodal metastasis or symptoms of systemic metastasis [30]. In contrast to unilateral cancers, the time interval between cancers in MBBC could affect prognosis [4,5]. This could explain our findings why in patients with a shorter time interval between cancers, nodal status of either cancer was predictive of metastatic staging outcomes (instead of the nodal status of the contralateral cancer alone), whereas in patients with a longer time interval between cancers, only the contralateral cancer nodal status was predictive.

There is little literature regarding the indication of metastatic staging for BBC diagnosed at an interval apart. We did not restrict it to MBBC but also included patients with DCIS too, since this is another entity also encountered clinically. Other strengths of our paper included that most patients had metastatic staging in our cohort, which allowed the identification of patients with systemic metastasis and the correlation of the need of metastatic staging in this group of patients. The data were also retrieved from a prospectively maintained database which has well-kept records of patients’ data.

Our paper was not without limitations. It was a retrospective single center study. However, a prospective study may be difficult to conduct since these BBCs, diagnosed at an interval apart, are uncommon. Being an uncommon entity, our sample size was inevitably small and our findings would need validation in larger studies. This group of patients would also qualify for genetic testing based on NCCN guidelines [31]. However, these data were not available for all patients.

## 5. Conclusions

BBC, diagnosed at an interval apart, is an uncommon entity. Nodal status combined with time interval between the cancers could possibly be useful to indicate a recommendation for metastatic staging at contralateral cancer diagnosis. This finding will require validation in future larger studies.

## Figures and Tables

**Table 1:** Characteristics of patients with ipsilateral invasive cancer and contralateral ductal carcinoma in situ (DCIS).

Characteristics	Primary Cancer N = 23 (%)	Contralateral Cancer N = 23 (%)
Age at diagnosis/years		
<50	12 (52.2)	5 (21.7)
>/=50	11 (47.8)	18 (78.3)
Histological features		
Invasive ductal cancer (IDC)	19 (82.6)	0 (0)
Invasive lobular cancer (ILC)	0 (0)	0 (0)
Others	4 (17.4)	0 (0)
Ductal carcinoma in situ (DCIS)	0 (0)	23 (100)
Grade *		NA
I	5 (21.7)	
II	9 (39.1)	
III	8 (34.8)	
Unknown	1 (4.4)	
Tumor size/mm *		NA
</=20	13 (56.5)	
>20–50	9 (39.1)	
>50	1 (4.4)	
Estrogen receptor (ER) *		NA
Positive	17 (73.9)	
Negative	6 (26.1)	
Progesterone receptor (PR) *		NA
Positive	14 (60.9)	
Negative	9 (39.1)	
Human epidermal growth factor receptor 2 (HER2) *		NA
Positive	2 (8.7)	
Negative	20 (87.0)	
Unknown	1 (4.3)	
Nodal status		
positive	8 (34.8)	0 (0)
negative	15 (65.2)	23 (0)

* Invasive cancer. NA—not applicable.

**Table 2:** Characteristics of metachronous bilateral breast cancer (MBBC) patients.

Characteristics	Primary Cancer N = 45(%)	Contralateral Cancer N = 45(%)
Age at diagnosis/years		
<50	18 (40.0)	7 (15.6)
>/=50	27 (60.0)	38 (84.4)
Histological features		
Invasive ductal cancer (IDC)	37 (82.2)	34 (75.6)
Invasive lobular cancer (ILC)	3 (6.7)	6 (13.3)
Others	5 (11.1)	5 (11.1)
Grade		
I	3 (6.7)	10 (22.2)
II	13 (28.9)	21 (46.7)
III	23 (51.1)	10 (22.2)
Unknown	6 (13.3)	4 (8.9)
Tumor size/mm		
</=20	20 (44.4)	31 (68.9)
>20–50	16 (35.6)	3 (6.7)
>50	4 (8.9)	1 (2.2)
Unknown	5 (11.1)	10 (22.2)
Estrogen receptor (ER) *		
Positive	30 (66.7)	34 (75.6)
Negative	15 (33.3)	10 (22.2)
unknown	0 (0)	1 (2.2)
Progesterone receptor (PR) *		
Positive	27 (60.0)	26 (57.8)
Negative	18 (40.0)	18 (40.0)
Unknown	0 (0)	1 (2.2)
Human epidermal growth factor receptor 2 (HER2)		
Positive	8 (17.8)	8 (17.8)
Negative	37 (82.2)	36 (80.0)
Unknown	0 (0)	1 (2.2)
Nodal status		
positive	18 (40.0)	12 (26.7)
negative	27 (60.0)	33 (73.3)

* Invasive cancer.

**Table 3:** Characteristics of patients with ipsilateral ductal carcinoma in situ (DCIS) and contralateral invasive cancer.

Characteristics	Primary Cancer N = 11 (%)	Contralateral Cancer N = 11 (%)
Age at diagnosis/years		
<50	4 (36.4)	4 (36.4)
>/=50	7 (63.6)	7 (63.6)
Histological features		
Invasive ductal cancer (IDC)	0 (0)	9 (81.8)
Invasive lobular cancer (ILC)	0(0)	0 (0)
Others	0(0)	2 (18.2)
Ductal carcinoma in situ (DCIS)	11 (100)	
Grade *	NA	
I		5 (45.4)
II		4 (36.4)
III		2 (18.2)
Tumor size/mm *	NA	
</=20		9 (81.8)
>20–50		2 (18.2)
>50		0 (0)
Estrogen receptor (ER) *	NA	
Positive		10 (90.9)
Negative		1 (9.1)
Progesterone receptor (PR) *	NA	
Positive		10 (90.9)
Negative		1 (9.1)
Human epidermal growth factor receptor 2 (HER2) *	NA	
Positive		0 (0)
Negative		11 (100)
Nodal status		
positive	0 (0)	2 (18.2)
negative	11 (100)	9 (81.8)

* Invasive cancer. NA—not applicable.

**Table 4:** Comparison of metachronous bilateral breast cancer (MBBC) patients with and without systemic metastasis at contralateral breast cancer diagnosis.

Characteristics N = 45	MBBC Patients with Systemic Metastasis N = 7 (%)	MBBC Patients without Systemic Metastasis N = 38 (%)	*p* Value
First cancer			
Age at diagnosis/years			0.4122
<50	4 (57.1)	14 (36.8)	
≥50	3 (42.9)	24 (63.2)	
Histological features			0.0507
Invasive ductal cancer	4 (57.1)	33 (86.8)	
Invasive lobular cancer	2 (28.6)	1 (2.6)	
others	1 (14.3)	4 (10.5)	
Grade			1.0000
I	0 (0)	3 (9.1)	
II	2 (33.3)	11 (33.3)	
III	4 (66.7)	19 (57.6)	
Unknown	1	5	
Tumor size/mm			0.1103
≤20	2 (40.0)	18 (51.4)	
>20–50	1 (20.0)	15 (42.9)	
>50	2 (40.0)	2 (5.7)	
Unknown	2	3	
Estrogen receptor (ER) *			0.6703
Positive	4 (57.1)	26 (68.4)	
Negative	3 (42.9)	12 (31.6)	
Progesterone receptor (PR)			1.0000
Positive	4 (57.1)	23 (60.5)	
Negative	3(42.9)	15 (39.5)	
Human epidermal growth factor receptor 2 (Her2)			0.0943
Positive	3 (42.9)	5 (13.2)	
Negative	4 (57.1)	33 (86.8)	
Nodal status			0.4122
positive	4 (57.1)	14 (36.8)	
negative	3 (42.9)	24 (63.2)	
Treatment details			0.3212
Mastectomy	7 (100.0)	30 (78.9)	
Lumpectomy	0 (0)	8 (21.1)	
Chemotherapy			1.0000
Yes	5 (71.4)	24 (63.2)	
No	2 (28.6)	14 (36.8)	
Radiotherapy			1.0000
Yes	3 (42.9)	17 (44.7)	
No	4 (57.1)	21 (55.3)	
Hormonal therapy			0.4329
Yes	3 (42.9)	23 (60.5)	
No	4 (57.1)	15 (39.5)	
Second cancer			
Age at diagnosis/years			0.2960
<50	2 (28.6)	5 (13.2)	
≥50	5 (71.4)	33 (86.8)	
Histological features			0.8160
Invasive ductal cancer	6 (85.7)	28 (62.2)	
Invasive lobular cancer	1 (14.3)	5 (11.1)	
Others	0 (0)	5 (11.1)	
Grade			0.2742
I	0 (0)	10 (28.6)	
II	5 (83.3)	16 (45.7)	
III	1 (16.7)	9 (25.7)	
Unknown	1	3	
Tumor size/mm			NA
≤20	-	31 (88.6)	
>20–50	-	3 (8.6)	
>50	-	1 (2.8)	
Unknown	7	3	
ER			1.0000
Positive	6 (85.7)	28 (75.7)	
Negative	1 (14.3)	9 (24.3)	
Unknown	0	1	
PR			1.0000
Positive	4 (57.1)	22 (59.5)	
Negative	3 (42.9)	15 (40.5)	
Unknown	0	1	
Her2			1.0000
Positive	1 (14.3)	7 (18.9)	
Negative	6 (85.7)	30 (81.1)	
Unknown	0	1	
Nodal status			0.0694
Positive	4 (57.1)	8 (21.1)	
Negative	3(42.9)	30 (78.9)	
Interval between cancers			0.1307
≤30 months	3 (42.9)	6 (15.8)	
>30 months	4 (57.1)	32 (84.2)	
Combined nodal status *			0.0322
Positive	5 (71.4)	10 (26.3)	
Negative	2 (28.6)	28 (73.7)	

* Defined as positive if either the first or contralateral cancer has nodal metastasis and diagnosed ≤30 months apart. If diagnosed >30 months apart, defined as positive only if contralateral cancer has nodal metastasis. NA—Not applicable since the patients with systemic metastasis did not undergo surgery.

## Data Availability

The data is available from the corresponding author upon request.
